# Unlocking the Role of Treg Cells Immune Response and Infectious Risk Following CAR T-Cell Therapy in Patients with Cancer

**DOI:** 10.3390/ijms26041602

**Published:** 2025-02-13

**Authors:** Destyn Dicharry, Alexandre E. Malek

**Affiliations:** Division of Infectious Diseases, Department of Medicine, School of Medicine, LSU Health Shreveport, Shreveport, LA 71103, USA; dld006@lsuhs.edu

**Keywords:** tregs, regulatory T-cells, CAR T-cell therapy, infections, cancer

## Abstract

Chimeric antigen receptor (CAR) T-cell therapy has brought hope for patients with cancer and showed promising results and a high cure rate in various types of hematological malignancies. However, cellular therapy can lead to profound immunodeficiency of the innate and adaptive immune systems, whether at the systemic or at the local cellular immune response, which is a major predisposing risk factor for invasive opportunistic infection, including fungal, viral, and bacterial pathogens. The role of regulatory T-cells (Tregs) and their antigen specificity in humans remains largely unknown, but Tregs have been implicated in a wide range of modulating viral and fungal infections. Though there have been many advancements regarding the use of CAR T-cells in treating hematological malignancies, the intricate and homeostatic role of Tregs in influencing therapeutic outcomes and infection risk remains underexplored. Most published literature on this topic focuses on the role of Treg in the immunosuppression necessary for successful CAR T-cell therapy rather than the dual function of Treg in immunosuppression and immune recovery. We intend to bridge this gap with a specific focus on the contribution of Tregs in the modulation of CAR T-cell efficacy and their role in opportunistic infections after therapy. In this review, we described the potential role and dynamics of Tregs following CAR T-cell therapy, offering an expanded understanding of their impact on patient outcomes and highlighting areas for future research.

## 1. Overview of CAR T-Cell Therapy and the Effects on the Host T-Cell

**Figure 1:**
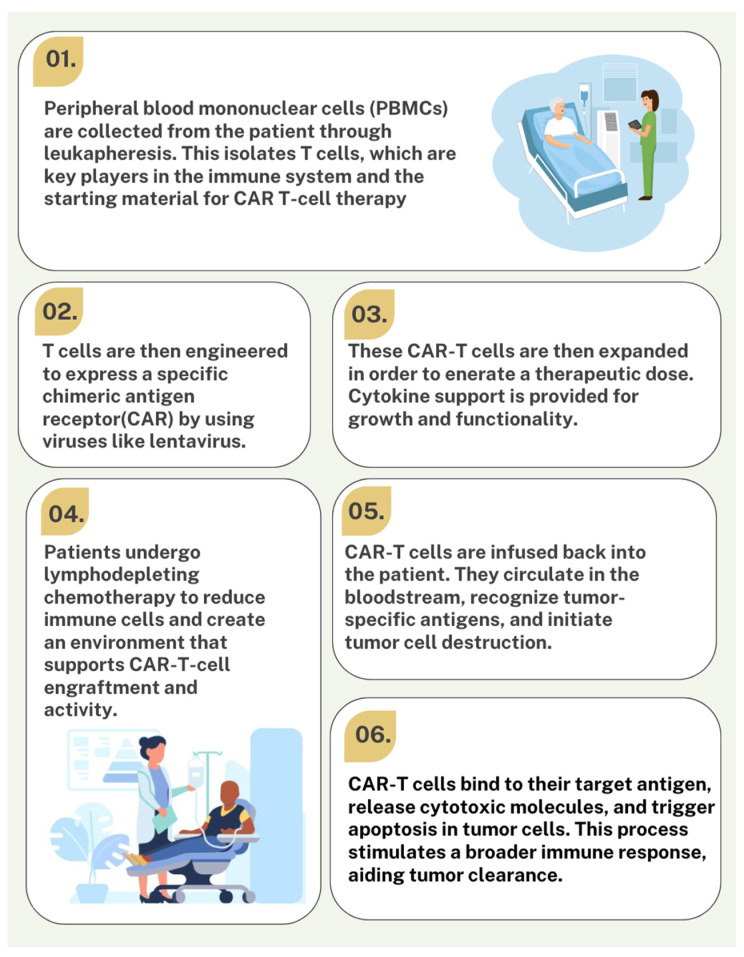
The steps illustrate the process of beginning CAR T-cell therapy.

Chimeric antigen receptor (CAR) T-cell therapy showed immense growth in advancing cancer treatment. The process involves collecting the patient’s T-cells, genetically modifying them to express a synthetic receptor that recognizes cancer cells and reintroducing these reprogrammed and engineered cells into the patient. The process begins with collecting peripheral blood mononuclear cells (PBMCs) from the patient through leukapheresis (Figure 1). The isolated T-cells are then genetically modified to express a chimeric antigen receptor (CAR) and expanded to a therapeutic dose [1]. The CAR T-cell is designed to bind specific tumor antigens, enabling T-cells to attack and destroy cancer cells. Prior to CAR T-cell infusion, it is necessary to administer lymphodepleting chemotherapy to reduce the host immune cells, modify the tumor microenvironment, and augment CAR T-cell expansion and the antitumor efficacy of the transferred T-cell [2].

The preparative regimen significantly reduces CD4 and CD8 T-cells, and following the CAR T-cell infusion, cytokine release syndrome (CRS) and immune effector cell-associated neurotoxicity syndrome (ICANS) might occur and require the use of tocilizumab and/or corticosteroids or other immunosuppressive therapies to control these severe toxicities. These therapeutic strategies further damage T-cell populations and delay cellular recovery [2]. The lymphodepleting chemotherapy also damages the thymus and the T-cell pool [3]. All of these are aspects of CAR T-cell therapy that can delay CD4+ T-cell recovery. The recovery of the adaptive immunity is slow and can take two years or more, contributing to prolonged immune vulnerability to opportunistic infections.

Recently, innovative epigenetics have been used to evaluate a patient’s response to CAR T-cell therapy. One study reports that a patient’s clinical benefit from CAR T-cells directed against CD19 (CART19) can be predicted by analyzing the preinfusion CART19 cell’s DNA methylation landscape [4]. There are now epigenetic biomarker libraries including DNA methylation-based immune profiling which has the potential to improve cell characterization, tracking, and patient response prediction in relation to CAR T-cell therapy [5]. There is still a need for identification of specific disease and cell epigenetic markers to ensure predictive models are as accurate as possible [6]. Innovations such as these will hopefully aid in increasing successful CAR T-cell therapeutic treatments.

## 2. The Dynamics of T-Regulatory Cells and CAR T-Cell Therapy

### 2.1. T-Regulatory Cells (Tregs)

A subset of CD4+ T-cells is affected by the CAR T-cell therapy process called Tregs, characterized by the expression of the transcription factor FOXP3. These cells are critical in maintaining immune homeostasis and preventing autoimmune responses by suppressing the activity of effector T-cells [7]. Tregs modulate the immune response of CD4+ and CD8+ T-cells, B-cells, NK cells, and dendritic cells through various mechanisms and are known to be reduced in number and function in autoimmune diseases. There are established epigenetic determinants of Treg function including the hypomethylation of Treg-specific demethylated region stabilizing FOXP3 expression and the DMA methyltransferases DNMT1 and UHR1 aiding in the maintenance of the Treg-specific methylation pattern to ensure Treg stability [8]. Tregs also contain the Satb1 chromatin organizer, which helps form chromatin loops. This enables interaction between enhancers and promoters, which stabilizes Treg identity [8]. The current evidence and findings demonstrated that immunosuppression is mediated by the secretion of anti-inflammatory cytokines (IL-10, IL-35, TGF-B), expression of inhibitory receptor CTLA-4, cytotoxic pathways (granzyme/perforin), modulating antigen-presenting cell (APC) function, and metabolic disruption through the utilization of adenosine and cAMP to inhibit effector cells [7].

### 2.2. The Role of T-Regulatory Cells (Tregs) in CAR T-Cell Therapy

Since Treg cells belong to a subset of specialized CD4+ T-cells that act as key modulators of immune responses, balance pathogen control, dampen the effects of an exaggerated immune response, and reduce the risk of end-organ tissue damage [7]. Tregs are a heterogenous subgroup of T-cells with several subpopulations that have been identified that consist of conventional thymic (tTregs), peripheral (pTregs), and tissue-specific Tregs such as bone marrow [9]. The nature of Treg cells has brought to light the importance of enhancing or depleting Tregs to optimize immune responses against infection or autoimmunity. These mechanisms of immunosuppression help mediate tissue injury but may also inhibit protective immune responses to pathogens and vaccines [10]. It has been purported that Tregs can also hinder effective immune responses against cancerous cells. CAR T-cell therapy is a revolutionary anti-cancer treatment that has ushered oncological therapy into a potentially curative era. The infused CAR T-cells combat malignancies by triggering T-cell cytotoxicity response via cytokine release and apoptosis induction [11]. In the context of CAR T-cell therapy, Tregs can inhibit therapeutic efficacy by creating an immunosuppressive tumor microenvironment as they produce immunosuppressive cytokines such as IL-10 and TGF-β. This can hinder CAR T-cell activation and proliferation and limit CAR T-cells’ cytotoxic activity. This has raised concerns that Tregs can lead to suboptimal therapeutic anti-cancer benefits and might have an impact on survival outcomes.

**Figure 2:**
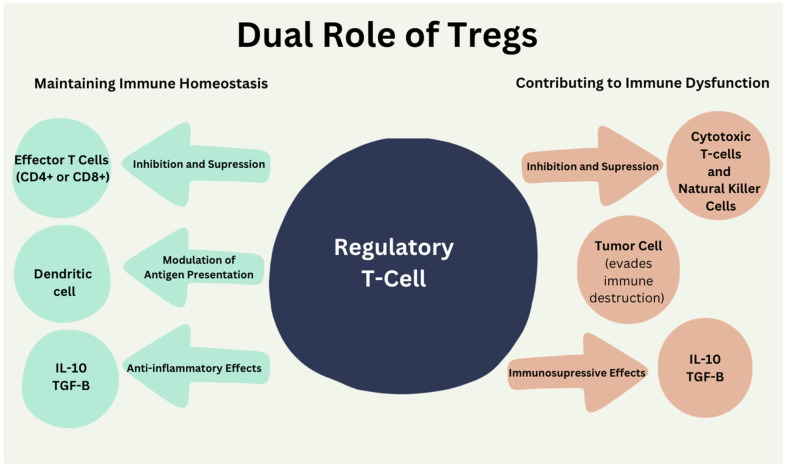
Tregs play a dual role in immune homeostasis.

While the lymphodepleting chemotherapy regimen before CAR T-cell therapy, initially reduces Treg populations, their eventual recovery and impact on tumor cells have not been clearly elucidated in the literature yet. Still, they may contribute to tumor tolerance and immune suppression [12]. Conversely, insufficient Treg cells recovery has been linked to a heightened risk of autoimmune disease [13]. Therapeutic strategies such as Treg depletion, checkpoint blockade immunotherapy, and tumor microenvironment modulation aim to optimize CAR T-cell therapy outcomes by mitigating Treg-related effects. Balancing these approaches is critical to enhancing CAR T-cell therapy effectiveness without disrupting immune equilibrium [14]. In contrast, in bone marrow transplant recipients, utilizing tissue-specific Tregs may have an adjunctive role of immunosuppressive therapy in reducing the risk of graft-versus-host disease and the number of immunosuppressive treatments required, and improving bone marrow engraftment (Figure 2) [15]. This highlights the dual role of Tregs. The roles can be described as (1) maintaining immune balance by suppressing excessive inflammation and preventing autoimmunity through mechanisms such as cytokine production (e.g., IL-10, TGF-β), and modulation of antigen-presenting cells (APCs); and (2) limiting antitumor immunity by creating an immunosuppressive tumor microenvironment, suppressing cytotoxic T-cells, and promoting tumor immune evasion. The functions of Tregs are complex and opposing, which emphasizes their impact on therapeutic outcomes and infection risks in CAR T-cell therapy as Tregs are critical cells in immune homeostasis [16] and can play a role in cancer progression (Figure 2).

### 2.3. Treg Cells Immune Reconstitution

**Figure 3:**
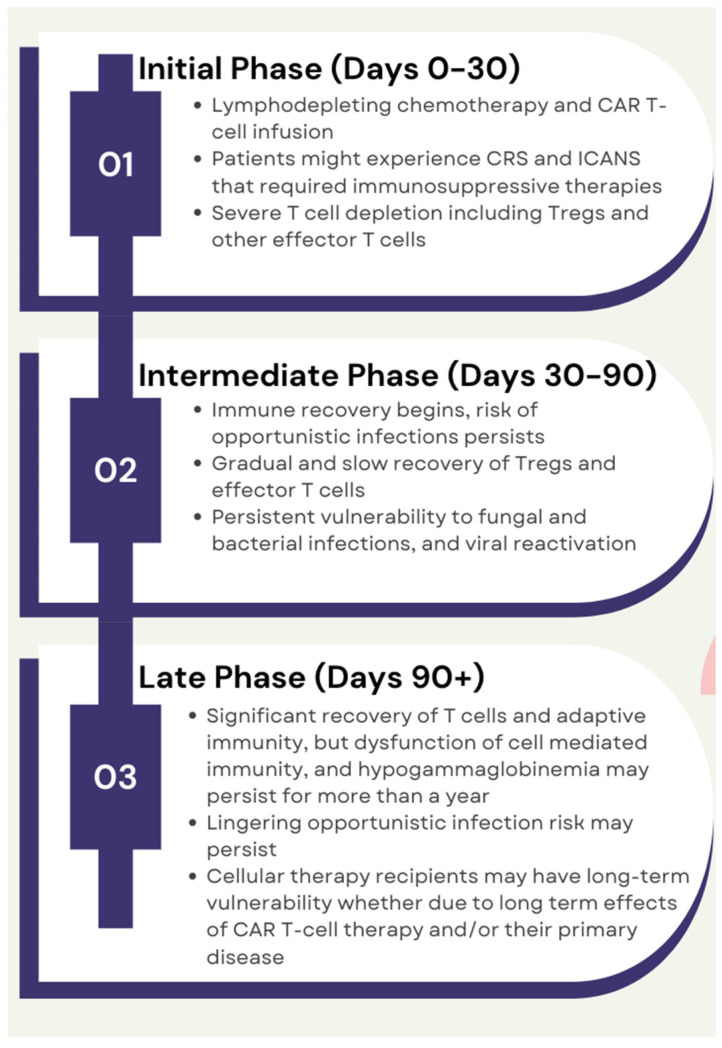
The timeline illustrates the phased recovery of the immune system following CAR T-cell therapy.

The lymphodepletion treatment before CAR T-cell therapy leads temporarily to severe T-cell immunodeficiency, including Tregs, and can persist for over a year. After CAR T-cell therapy, the immune system undergoes substantial changes, and the recovery is delayed and multifaceted. The Initial Phase (Days 0–30) can be characterized by severe lymphodepletion, CRS, and ICANS. During the Intermediate Phase (Days 30–90)**,** gradual recovery of Tregs and effector T-cells begins, but patients remain vulnerable to opportunistic infections such as bacterial, viral, and fungal pathogens. By the Late Phase (Day 90+), immune homeostasis is largely restored, with the recovery of adaptive immunity and Treg-mediated balance. These changes in immune function emphasize the role of Tregs throughout the recovery process [16]. Over time, the recovery of T-cell immunity relies mainly on peripheral memory T-cell expansion, and Tregs tend to repopulate, potentially re-establishing their immunosuppressive roles. The recovery rate and extent depend on several factors such as patient health, tumor burden, and CAR T-cell design (Figure 3). Post-therapy Tregs may exhibit altered phenotypes or functions due to immune modulation induced by CAR T-cells [17]. Of note, this complex T-cell recovery depends on the interaction of T-cell progenitors with the host thymic epithelium. Moreover, this process can be affected by various factors that could occur following cell infusion, including the development of CRS or ICANS and other cellular therapy-related toxicities that, in many situations, require the use of glucocorticoids, steroids-sparing immunomodulators, and biologic therapies.

### 2.4. Complications Related to Tregs Post-CAR T-Cell Therapy

Tregs might contribute to both positive and negative outcomes after CAR T-cell therapy. While Tregs help restore immune balance, excessive Treg activity can dampen anti-tumor effects [16]. Tumor cells may exploit Treg-mediated suppression to evade immune detection, leading to disease relapse or immune tolerance. In a recent study, authors explored how normal immune cell reconstitution impacts progression-free survival, overall survival, and risks of adverse events like infections or secondary cancers in chronic lymphocytic leukemia (CLL) patients. They found that Tregs in the CLL microenvironment have been shown to promote CLL survival and immune evasion. High CD4+ T-cell counts after lymphodepletion may indicate a reconstitution of these supportive immune properties, which could facilitate relapse [18].

In addition, it has been demonstrated that early CD4+ T-cell recovery following CAR T-cell infusion may indicate CAR T-cell therapy dysfunction rather than effective immune reconstitution. This could reflect suboptimal lymphodepleting chemotherapy or underlying CAR T-cell resistance mechanisms. Delayed CD4+ T-cell recovery aligns with better outcomes, suggesting the need for careful monitoring and tailored interventions based on early immune reconstitution patterns [16]. Researchers found that early recovery of CD4+ T-cells at Day 30+ after CAR T-cell therapy was associated with worse overall survival and progression-free survival in diffuse large B-cell lymphoma patients (HR = 4.47, *p* = 0.03). Tumor relapse occurred in 82% of patients with early CD4+ T-cell recovery versus 47% in those without [16]. Although early recovery was associated with worse cancer outcomes, prolonged CD4+ T-cell lymphopenia was linked to severe life-threating infections such as viral reactivations and invasive mold infections. In addition, studies have shown a regulatory role of type 2 CD8+ T suppressors cells in modulating immune cells hemostasis and protecting against the development of chronic inflammatory disease and the cells’ potential role in a tumor microenvironment [19].

### 2.5. Comorbid Conditions and Immune Recovery After CAR T-Cell Therapy

Medical comorbidities such as cardiac, pulmonary, and psychiatric underlying conditions may have a critical role in predicting early mortality. In one study, researchers investigated the impact of medical comorbidities on patients with relapsed/refractory large B-cell lymphoma. Cardiac comorbidities were found to significantly increase the 100-day non-relapse mortality (NRM), experiencing a 26.9% NRM compared to 8.1% for those without cardiac conditions. Severe hepatic dysfunction also posed a significant increased risk, exhibiting a 50% higher 100-day NRM compared to 8.9% for those patients without liver complications. Psychiatric disturbances, including anxiety or depression that required treatment, also displayed an increased 100-day NRM of 19.6% compared to 7.8% for patients who did not experience these conditions. Patients > 60 of age showed significantly higher mortality rates than younger patients within the first 100 days post-therapy [20]. Comorbid conditions such as cardiac and liver dysfunction may exacerbate immune homeostatic disruption. This may impact the recovery and function of Tregs post-therapy. Since Tregs are essential for mitigating excessive immune responses such as those exhibited in the potentially fatal CRS, heart, and liver issues that contribute to systemic inflammation, Treg recovery may be hindered. This would leave patients with comorbidities more susceptible to immune-related toxicities and infections.

A study investigating the mechanisms of heart disease and Treg cells stated that since Tregs have such a critical role in immune regulation and inflammation, their disruption can lead to rogue immune responses which may lead to pathological changes. They also highlight a central role of Tregs in atherosclerosis, hypertension, myocardial infarction and remodeling, myocarditis, dilated cardiomyopathy, and heart failure [21]. In addition, they observed that enhancement of inflammatory reactions was observed in the absence of Tregs, which emphasizes their integral role in maintaining cardiovascular health. Adding another layer to Tregs’ role in how comorbid conditions affect CAR T-cell therapy, this study shines light on the importance of recognizing and understanding the complex nature of Treg cells.

This same concept is observed in liver dysfunction. One study found that Treg deficiency can have significant effects on the liver such as exacerbation of inflammatory injury in nonalcoholic fatty liver disease, autoimmune hepatitis, primary biliary cholangitis, and acute liver transplant rejection. Depletion of Tregs is also linked to anxiety and depression. A study using a Treg-depleted mice model found that transient depletion of Foxp3-expressing cells (Tregs) may influence anxiety or depression-like behaviors. They stated that this is potentially due to inflammatory response and immune cell trafficking in the hippocampal formation of the Treg-depleted mice, as the hippocampal formation is strongly linked to anxiety and depression-like behaviors in rodents [22]. Tregs’ vast involvement in a variety of different comorbidities known to impact CAR T-cell therapy mortality should be a focus of future research, as the depletion of Tregs from this therapy may have many unexplored effects outside of the most commonly researched, like ICANS and CRS.

## 3. Infectious Complications Post-CAR T-Cell Therapy

Infections are a prominent complication of CAR T-cell therapy, driven by immune suppression and therapy-related factors. During the early phase of infection (days 0–30), bacterial infections predominate due to neutropenia induced by lymphodepleting chemotherapy. Common bacterial pathogens include gram-positive and gram-negative organisms such as *Streptococcus* species, *Clostridium difficile*, and *Enterobacteriaceae*. During the late phase of infection (day 30+), viral infections, primarily respiratory viruses, become more common due to sustained lymphopenia and hypogammaglobulinemia. Herpesvirus reactivation (e.g., cytomegalovirus [CMV]) and opportunistic invasive fungal infections are noted during this phase and are increasingly reported following CAR T-cell therapy in patients with hematologic malignancies [23].

### 3.1. Cytomegalovirus Infection

CMV reactivation is a growing issue reported following CAR T-cell therapy with a high disease burden in CAR T-cell therapy recipients, especially in the absence of robust data to guide optimal clinical monitoring, preventive measures, and management [24]. Treg’s role in the prevalence of opportunistic infections in an immunosuppressed state is emphasized through a study investigating the contributions of CMV-specific Treg and T-cell effector subsets to CMV end-organ disease in HIV-infected individuals. The study investigators discovered a potentially detrimental role for CMV-specific Treg in patients with advanced HIV infection. By suppressing effective immune responses to CMV, Treg cells may facilitate progression to CMV end-organ disease [25]. Authors demonstrated that excessive Tregs immune suppression can undermine defense mechanisms. However, several scenarios were postulated that Treg cells could survive longer compared to T-cell effectors in chronic HIV infection.

### 3.2. Herpesvirus

Herpetic infections are a cause for concern in individuals who have received CAR T-cell therapy due to potential B-cell depletion and hypogammaglobulinemia and the overall profound immunosuppression that comes along with a successful treatment. Human herpesvirus 6B (HHV-6B) reactivation and disease have been reported as increasing in number, which raised questions about incidence and outcomes of HHV-6B infection after CAR T-cell therapy, and routine monitoring was called into question [26]. One study found that out of 89 individuals who received CAR T-cell therapy who were monitored for 12 weeks post-infusion, HHV-6B reactivation occurred in only eight patients. They interpret this as HHV-6B reactivation being infrequent and not requiring monitoring [27]. CMV and HSV have also been reported to cause pneumonia in individuals with CRS from CAR T-cell therapy. A study reporting and analyzing these cases attributes the increased severity to the subsequent reactivation of CMV and HSV-1, which ultimately lead to respiratory failure and death [28]. This highlights the growing threats of CMV and HSV to cause fatal pneumonia in CAR T-cell recipients and the need for consideration of herpesviruses when dealing with end-organ disease after CAR T-cell therapy. The presence of intense viral reactivation in CAR T-cell therapy recipients is closely related to severe immunosuppression, including the depletion of Tregs. The individuals discussed above experienced exaggerated immune responses in the form of CRS, with the depletion of Tregs being a leading cause of this reaction. Since functional Tregs are typically low post-CAR T-cell therapy, the exaggerated inflammatory response of CRS may have further compromised the patient’s ability to manage viral reactivation.

### 3.3. Epstein-Barr Virus (EBV)

The role of EBV infection in mediating lymphoproliferative diseases has been well documented in the literature to occur as a complication of organ transplants or hematopoietic stem cell transplantation, but there have also been cases reported after CAR T-cell therapy [29]. One study reported that two patients experienced this phenomenon. The recommended treatment for EBV-associated lymphoproliferative disorders post-CAR T-cell therapy can be immune checkpoint inhibitors or B-cell depleting agents [29]. In the case of EBV-associated lymphoproliferative disorders, the depletion of Tregs may have allowed for the unchecked proliferation of EBV-infected B-cells [30]. Tregs play a crucial role in EBV latency and preventing its reactivation. When functional Tregs are diminished, EBV-infected B-cells can escape immune surveillance. This can then progress to uncontrolled cell proliferation and the development of EBV-associated lymphoproliferative disorders.

The severely weakened adaptive immunity post-CAR T-cell therapy is compounded by a delayed recovery of Tregs, which play a pivotal role in the immune response to these opportunistic infections. Since they are essential to preventing excessive inflammation, their delayed recovery post-CAR T-cell therapy can lead to an inadequate balance between controlling viral replication and avoiding tissue damage. The case studies presented above call for increased and careful monitoring as well as early interventions should these infections arise in this vulnerable population.

### 3.4. Coronavirus Disease 2019 (COVID-19)

The COVID-19 pandemic affected people’s healthcare in many ways, including those who have received CAR T-cell therapy for B-cell malignancies. One study investigated COVID-19 infection’s impact on this population and found that 80% of the 56 individuals analyzed who received CAR T-cell therapy required hospitalization due to COVID-19, with a median stay of 26.5 days. Around 49% were admitted to the intensive Care Unit (ICU) and 73% of ICU patients required invasive ventilation. The mortality attributable to COVID-19 was 41% and the study found increased age and other pre-existing medical comorbidities as significant independent risk factors for mortality in patients who received CAR T-cell therapy. They also found that neither the elapsed time since CAR T-cell infusion nor a history of ICANS or CRS proved significant in impacting outcomes [31].

This highlights that the profound immunosuppression in CAR T-cell therapy recipients can have lasting and potentially fatal outcomes when met with COVID-19 infection. This is likely driven by B-Cell depletion, hypogammaglobulinemia, and ongoing cytopenias, Treg cells may also play a multi-faceted role in increased severity of COVID-19 infections in CAR T-cell therapy recipients. Severe COVID-19 can result in cytokine release syndrome, which is attributable to depletion of Tregs as preventing inflammation-induced injury caused by the infection [32].

### 3.5. Fungal Infection

As discussed, Tregs prevent exuberant immune responses that can damage the host vital organs. Bacher and colleagues discovered that expanding antigen-specific Tregs against mucosal *Aspergillus fumigatus* and *Candida albicans* antigens is essential to modulate the immune responses. In support of this, authors found that in patients with significant allergic reactions to *Aspergillus fumigatus*, the T-cell response is mainly predominated by memory T-cell response as opposed to Tregs response, and this was also found in patients with respiratory tract diseases such as patients with cystic fibrosis [33]. Of note, immunocompromised patients such as cellular-therapy recipients are continuously challenged by the exposure to various fungal pathogens, which are either present in the environment or part of the commensal microbiota and can have a life-threatening invasive fungal infection or hypersensitivity reaction to fungal spores due to the alteration of Tregs function and other immune T-helper cells.

Strikingly, a study revealed that after bone marrow transplantation, *Aspergillus*-specific T-cell response was notable for low IFN-g/IL-10 ratio, which indicated that a T_H_2 cell response could potentially prolong the risk for having invasive aspergillosis in contrast to the protective role of T_H_1-cell response. As is known, the traditional risk factors for invasive Aspergillosis are mainly neutropenia and phagocyte dysfunction, but more recently, T-cells have been shown to play a role in host defense in controlling *Aspergillus* infections [34]. In light of the growing role of type 1 T-cell cytokines in fungal infection, a group of scientists developed and validated a novel approach to generate anti-*Aspergillus* CAR T-cell therapy as a promising adjunctive treatment modality against recalcitrant invasive fungal infection [35,36].

Another dimension of immunosuppression is the presence of hypogammaglobulinemia and prolonged cytopenia, which contribute to a higher risk of infection post-CAR T-cell therapy, even beyond 90 days post-infusion. Respiratory infections are the most observed, as well as opportunistic, fungal infections [37]. In addition, patients undergoing CAR T-cell therapy are at heightened risk of severe COVID-19 infection due to immune suppression with prolonged viral clearance observed in some cases [17].

Some other common challenges that arise post-CAR T-cell therapy are CRS and ICANS. Lu and colleagues found that CRS and ICANS affected 56% and 21% of individuals who received the first approved CAR T-cellular therapy [11]. As for CRS, though not directly caused by Tregs, their suppressive functions may interact with the inflammatory milieu of CRS, complicating its resolution. However, insufficient Treg recovery may result in a loss of immune tolerance [17]. For example, patients can develop autoimmune pneumonitis, colitis, and/or thyroiditis [37]. This again highlights the homeostatic role of Tregs even in T-cell recovery post-CAR T-cell therapy. It has been studied that targeting the Wnt/B-catenin pathway can suppress immunosuppressive Treg responses, sustaining Th-1-mediated protective immunity, particularly against invasive fungal infections [38]. This could be useful in managing immune homeostasis peri- and after CAR T-cell therapy. It could improve the tumor microenvironment’s immune permissiveness, minimize off-target effects, and prevent opportunistic fungal infections.

## 4. Conclusions

Tregs play a complex role following CAR T-cell therapy, acting as guardians of immune balance and obstacles to therapeutic efficacy. Understanding and modulating Treg dynamics is crucial for enhancing CAR T-cell therapy outcomes and minimizing infectious complications. Further research is needed to optimize targeting Tregs without compromising immune homeostasis. Elucidating the exact therapeutic role of Tregs post-CAR T-cell therapy and its clinical effects requires further preclinical and clinical studies and highlighting of the role of antigen specificity of Tregs and their protective roles.

## 5. Future Research and Directives

Unlocking the unique role and dynamics of regulatory T-cells in greater depth is an unmet need for CAR T-cell therapy recipients and patients with cancer. There is a necessity for further explanation of the long-term immune consequences of CAR T-cell therapy and its specific alterations to immune dynamics in order to optimize therapeutic outcomes and minimize complications. These investigations should specifically focus on the dual role of Tregs in suppressing excessive immune responses and promoting immune recovery. Studies which aim to identify parameters to predict and manage immune recovery and infection risk in CAR T-cell therapy patients are highly needed. In addition, new targeted therapeutic approaches at balancing Tregs modulation without compromising immune function are also critical. The more that these gaps are addressed, a clearer path can be paved for safer administration of and recovery from CAR T-cell therapies.

## References

[1] Kenderian S.S., Porter D.L., Gill S. (2017). Chimeric Antigen Receptor T Cells and Hematopoietic Cell Transplantation: How Not to Put the CART Before the Horse. Biol. Blood Marrow Transplant..

[2] Puckrin R., Jamani K., Jimenez-Zepeda V.H. (2024). Long-term survivorship care after CAR-T cell therapy. Eur. J. Haematol..

[3] Velardi E., Tsai J.J., van den Brink M.R.M. (2021). T cell regeneration after immunological injury. Nat. Rev. Immunol..

[4] Garcia-Prieto C.A., Villanueva L., Bueno-Costa A., Davalos V., González-Navarro E.A., Juan M., Urbano-Ispizua Á., Delgado J., Ortiz-Maldonado V., del Bufalo F. (2022). Epigenetic Profiling and Response to CD19 Chimeric Antigen Receptor T-Cell Therapy in B-Cell Malignancies. JNCI J. Natl. Cancer Inst..

[5] Salas L.A., Zhang Z., Koestler D.C., Butler R.A., Hansen H.M., Molinaro A.M., Wiencke J.K., Kelsey K.T., Christensen B.C. (2022). Enhanced cell deconvolution of peripheral blood using DNA methylation for high-resolution immune profiling. Nat. Commun..

[6] Benincasa G., Strozziero M.G., Di Pastena M.A., Criscuolo C., Cetani G., Trama U., Napoli C. (2024). Epigenetic challenges on the horizon of chimeric antigen receptor-T. Cytotherapy.

[7] Sanchez A.M., Yang Y. (2011). The role of natural regulatory T cells in infection. Immunol. Res..

[8] Joudi A.M., Reyes Flores C.P., Singer B.D. (2022). Epigenetic Control of Regulatory T Cell Stability and Function: Implications for Translation. Front. Immunol..

[9] Miyara M., Yoshioka Y., Kitoh A., Shima T., Wing K., Niwa A., Parizot C., Taflin C., Heike T., Valeyre D. (2009). Functional delineation and differentiation dynamics of human CD4+ T cells expressing the FoxP3 transcription factor. Immunity.

[10] Stephen-Victor E., Bosschem I., Haesebrouck F., Bayry J. (2017). The Yin and Yang of regulatory T cells in infectious diseases and avenues to target them. Cell Microbiol..

[11] Lu J., Jiang G. (2022). The journey of CAR-T therapy in hematological malignancies. Mol. Cancer.

[12] Golubovskaya V., Wu L. (2016). Different subsets of T cells, memory, effector functions, and CAR-T immunotherapy. Cancers.

[13] Dominguez-Villar M., Hafler D.A. (2018). Regulatory T cells in autoimmune disease. Nat. Immunol..

[14] Arjomandnejad M., Kopec A.L., Keeler A.M. (2022). CAR-T Regulatory (CAR-Treg) Cells: Engineering and Applications. Biomedicines.

[15] Ashman J., Mutsonziwa N., Romano M., Kordasti S., Lombardi G., Shangaris P. (2023). Regulatory T cell niche in the bone marrow, a new player in Haematopoietic stem cell transplantation. Blood Rev..

[16] Gambella M., Carlomagno S., Mangerini R., Colombo N., Parodi A., Ghiggi C., Giannoni L., Coviello E., Setti C., Luchetti S. (2024). Early CAR− CD4+ T-lymphocytes recovery following CAR-T cell infusion: A worse outcome in diffuse large B cell lymphoma. eJHaem.

[17] Wudhikarn K., Perales M.A. (2022). Infectious complications, immune reconstitution, and infection prophylaxis after CD19 chimeric antigen receptor T-cell therapy. Bone Marrow Transplant..

[18] Gauthier M., Durrieu F., Martin E., Peres M., Vergez F., Filleron T., Obéric L., Bijou F., Mary A.Q., Ysebaert L. (2019). Prognostic role of CD4 T-cell depletion after frontline fludarabine, cyclophosphamide and rituximab in chronic lymphocytic leukaemia. BMC Cancer.

[19] Filaci G., Rizzi M., Setti M., Fenoglio D., Fravega M., Basso M., Ansaldo G., Ceppa P., Borgonovo G., Murdaca G. (2005). Non-antigen-specific CD8(+) T suppressor lymphocytes in diseases characterized by chronic immune responses and inflammation. Ann. N. Y. Acad. Sci..

[20] Tang K., Khwaja R., Feng L., Strati P., Steiner R.E., Nair R., Flowers C.R., Saini N., Ramdial J.L., Srour S.A. (2022). Comorbidities Associated with Early Mortality after CD19 CAR-T Cell Therapy. Blood.

[21] Xia Y., Gao D., Wang X., Liu B., Shan X., Sun Y., Ma D. (2024). Role of Treg cell subsets in cardiovascular disease pathogenesis and potential therapeutic targets. Front. Immunol..

[22] Yang E.J., Al Rahim M., Griggs E., Iban-Arias R., Pasinetti G.M. (2023). Transient anxiety-and depression-like behaviors are linked to the depletion of Foxp3-expressing cells via inflammasome in the brain. PNAS Nexus.

[23] Kampouri E., Little J.S., Rejeski K., Manuel O., Hammond S.P., Hill J.A. (2023). Infections after chimeric antigen receptor (CAR)-T-cell therapy for hematologic malignancies. Transpl. Infect. Dis..

[24] Mihalić A., Železnjak J., Lisnić B., Jonjić S., Juranić Lisnić V., Brizić I. (2024). Immune surveillance of cytomegalovirus in tissues. Cell. Mol. Immunol..

[25] Weinberg A., Bosch R., Bennett K., Tovar-Salazar A., Benson C.A., Collier A.C., Zolopa A., Gulick R.M., Wohl D., Polsky B. (2014). Regulatory T cells and the risk of CMV end-organ disease in patients with AIDS. J. Acquir. Immune Defic. Syndr..

[26] Peggs K.S. (2024). Human herpesvirus 6 and CAR T-cell toxicity. Blood.

[27] Kampouri E., Krantz E.M., Xie H., Ibrahimi S.S., Kiem E.S., Sekhon M.K., Liang E.C., Cowan A.J., Portuguese A.J., Green D.J. (2024). Human herpesvirus 6 reactivation and disease are infrequent in chimeric antigen receptor T-cell therapy recipients. Blood.

[28] Heldman M.R., Ma J., Gauthier J., O’Hara R.A., Cowan A.J., Yoke L.M., So L., Gulleen E., Duke E.R., Liu C. (2021). CMV and HSV Pneumonia After Immunosuppressive Agents for Treatment of Cytokine Release Syndrome Due to Chimeric Antigen Receptor–modified T (CAR-T)-Cell Immunotherapy. J. Immunother..

[29] Zhang S., Zhou X., Zhang S., Wang N., Zhang T., Zhang D., Ao Q., Cao Y., Huang L. (2024). EBV-associated lymphoproliferative disease post-CAR-T cell therapy. Front. Med..

[30] Ahmed E.H., Lustberg M., Hale C., Sloan S., Mao C., Zhang X., Ozer H.G., Schlotter S., Smith P.L., Jeney F. (2023). Follicular Helper and Regulatory T Cells Drive the Development of Spontaneous Epstein–Barr Virus Lymphoproliferative Disorder. Cancers.

[31] Spanjaart A.M., Ljungman P., De La Camara R., Tridello G., Ortiz-Maldonado V., Urbano-Ispizua A., Barba P., Kwon M., Caballero D., Sesques P. (2021). Poor Outcome of Patients with COVID-19 after CAR T-Cell Therapy for B-Cell Malignancies: Results from a Multicenter Study on Behalf of the European Society for Blood and Marrow Transplantation (EBMT) Infectious Diseases Working Party and the European Hematology Association (EHA) Lymphoma Group. Blood.

[32] Wang Y., Zheng J., Islam M.S., Yang Y., Hu Y., Chen X. (2021). The role of cd4^+^foxp3^+^ regulatory t cells in the immunopathogenesis of COVID-19: Implications for treatment. Int. J. Biol. Sci..

[33] Bacher P., Kniemeyer O., Schönbrunn A., Sawitzki B., Assenmacher M., Rietschel E., Steinbach A., Cornely O.A., Brakhage A.A., Thiel A. (2014). Antigen-specific expansion of human regulatory T cells as a major tolerance mechanism against mucosal fungi. Mucosal Immunol..

[34] Hebart H., Bollinger C., Fisch P., Sarfati J., Meisner C., Baur M., Loeffler J., Monod M., Latgé J.-P., Einsele H. (2002). Analysis of T-cell responses to Aspergillus fumigatus antigens in healthy individuals and patients with hematologic malignancies. Blood.

[35] Kumaresan P.R., Wurster S., Bavisi K., da Silva T.A., Hauser P., Kinnitt J., Albert N.D., Bharadwaj U., Neelapu S., Kontoyiannis D.P. (2024). A novel lentiviral vector-based approach to generate chimeric antigen receptor T cells targeting *Aspergillus fumigatus*. mBio.

[36] Kumaresan P.R., da Silva T.A., Kontoyiannis D.P. (2017). Methods of Controlling Invasive Fungal Infections Using CD8+ T Cells. Front. Immunol..

[37] Chakraborty R., Hill B.T., Majeed A., Majhail N.S. (2021). Late Effects after Chimeric Antigen Receptor T Cell Therapy for Lymphoid Malignancies. Transplant. Cell Ther..

[38] Karnam A., Bonam S.R., Rambabu N., Wong S.S.W., Aimanianda V., Bayry J. (2021). Wnt-b-Catenin Signaling in Human Dendritic Cells Mediates Regulatory T-Cell Responses to Fungi via the PD-L1 Pathway. mBio.

